# CD8⁺ T Cell infiltration and spatial organization in cancer: mechanisms, barriers, and therapeutic strategies

**DOI:** 10.1186/s43556-026-00612-3

**Published:** 2026-10-10

**Authors:** Lylou Milian, Mannon Geindreau, Cassandre Pignol, Frédérique Vegran

**Affiliations:** Université Bourgogne Europe, Centre de Lutte Contre Le Cancer G-F. Leclerc, INSERM, CTM UMR 1231 [Equipe TIRECS], 21000 Dijon, France

**Keywords:** CD8^+^ T cells, Tumor infiltration, Tumor Microenvironment, Chemokines and chemokine receptors, T cell recruitment

## Abstract

The infiltration of tumors by CD8⁺ T lymphocytes is a critical determinant of antitumor immunity and response to immunotherapy. However, the presence of tumor-specific CD8⁺ T cells does not necessarily translate into effective tumor control, as multiple biological barriers can prevent their recruitment, spatial distribution, persistence, and functional activity. This review provides a comprehensive overview of the multistep mechanisms regulating CD8⁺ T-cell infiltration, from immune priming and systemic trafficking to vascular entry, intratumoral migration, and tissue residency. We discuss how alterations in chemokine networks, dysfunctional tumor vasculature, extracellular matrix remodeling, stromal barriers, and immunosuppressive signals collectively drive immune exclusion and resistance to therapy. Unlike previous reviews that have focused on individual aspects of T-cell trafficking, tumor immune contexture, or immune exclusion, we integrate these interconnected mechanisms as successive biological checkpoints and link them to therapeutic strategies aimed at overcoming specific barriers. These include restoration of antigen presentation and dendritic cell function, reprogramming of chemokine pathways, vascular normalization, stromal remodeling, innate immune activation, and induction of tertiary lymphoid structures. This integrated framework highlights why increasing CD8⁺ T-cell infiltration alone may be insufficient and supports the development of rational, sequential, and spatially guided combination strategies. Advances in spatial transcriptomics, single-cell technologies, and multiplex imaging are expected to enable personalized identification of dominant immune barriers and rational design of combination therapies. Ultimately, the goal is shifting from simply activating T cells toward engineering tumor ecosystems that support sustained CD8⁺ T-cell infiltration, organization, and effector function.

## Introduction

High CD8⁺ T cell density within the tumor microenvironment (TME) is associated with a favorable prognosis in cancers such as oral squamous cell carcinoma, colorectal cancer (CRC), breast cancer, lung cancer, and melanoma [1–5], whereas low infiltration correlates with immunosuppression and poor outcomes. However, even when present, CD8⁺ T cells may be functionally impaired, spatially excluded, or exhausted, highlighting a key paradox: tumor-specific T cells do not necessarily mediate an effective anti-tumor response.

CD8⁺ T cells are central to the efficacy of immune checkpoint inhibitors (ICIs) targeting PD-1, PD-L1, or CTLA-4 which aim to reinvigorate exhausted CD8⁺ T cells. Indeed, T-cell exhaustion arises during sustained antigen exposure and chronic inflammatory stimulation and is associated with a progressive remodeling of T-cell function and phenotype. Unlike transient effector dysfunction, exhaustion is characterized by stable transcriptional, epigenetic, metabolic, and functional alterations that progressively limit anti-tumor activity. Within the TME, exhaustion is reinforced by multiple environmental constraints, including persistent T cell receptor (TCR) stimulation, hypoxia, nutrient deprivation, metabolic stress, and immunosuppressive cytokines. ICIs effectiveness depends on the presence of tumor antigen-specific CD8⁺ T cells, leading to the classification of “hot” tumors, characterized by high CD8⁺ T cell infiltration and generally better responses to ICIs, and “cold” tumors, which are poorly infiltrated by immune cells. CD8⁺ T cell density and functional state are therefore key predictive biomarkers [6]. A third phenotype, “immune-excluded” tumors [7], is characterized by T cells located in the vicinity of the tumor or within the stroma but failing to penetrate the tumor parenchyma due to physical and biological barriers such as dense extracellular matrix (ECM), immunosuppressive signaling, or altered chemotaxis [8].

Spatial organization of immune cells is also critical for anti-tumor immunity. CD8⁺ T cells can integrate into highly organized lymphoid aggregates known as tertiary lymphoid structures (TLS) [9, 10]. These ectopic lymphoid structures resemble lymph nodes, develop in chronically inflamed tissues, and reflect an immunologically active microenvironment [11, 12]. In the tumor context, TLS are associated with enhanced effector T cell infiltration, local antigen priming, clonal expansion and stronger CD8⁺ cytotoxic responses. Clinically, TLS presence correlates with better prognosis across multiple cancer types, and with improved responses to immunotherapies [13, 14].

Throughout this Review, we propose a framework that differs from previous reviews by considering CD8^+^ T cell infiltration not as a single even, but as a multistep process in which successive biological checkpoints determine whether tumor-reactive T cells can ultimately reach and function within the tumor. We integrate these interconnected steps – from T-cell generation and systemic trafficking to tumor entry, intratumoral positioning, spatial organization, persistence, and effector function – to identify the specific barriers that can limit effective antitumor immunity at each stage. Importantly, we map therapeutic strategies onto these individual checkpoints and discuss how their rational combination may overcome barriers that cannot be addressed by increasing T-cell infiltration alone.

## The multistep biology of CD8⁺ T-cell recruitment into tumors

**Fig. 1:**
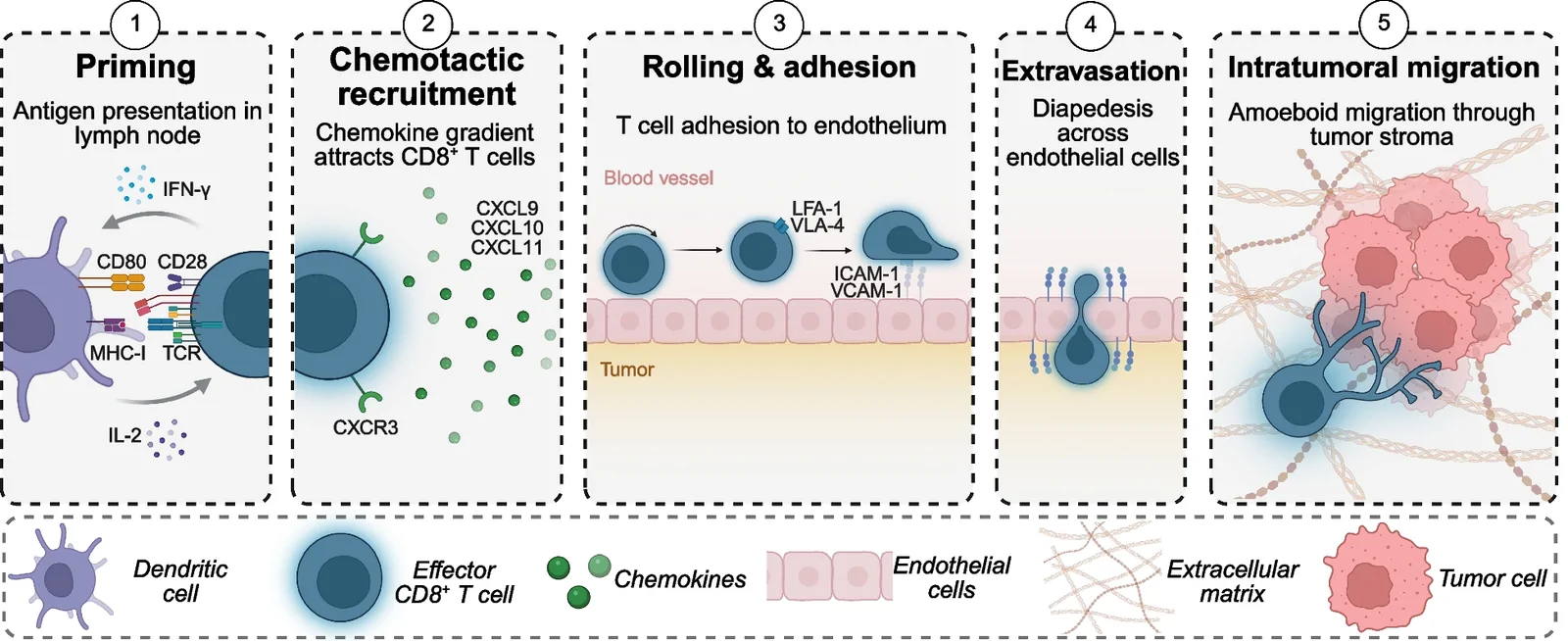
Multistep process governing CD8⁺ T-cell recruitment and infiltration into tumors. Efficient accumulation of tumor-reactive CD8⁺ T lymphocytes within the TME requires a coordinated sequence of biological events, including priming, systemic trafficking, vascular entry, and intratumoral migration. (1) Priming: Tumor antigens are captured and presented by DCs in lymphoid organs, leading to CD8⁺ T-cell activation through TCR engagement, co-stimulatory signals (CD80/CD28), and cytokine-mediated differentiation. (2) Chemotactic recruitment: Activated effector CD8⁺ T cells migrate toward tumors following chemokine gradients, particularly through CXCR3 recognition of CXCL9, CXCL10, and CXCL11. (3) Rolling and adhesion: T cells interact with the tumor vasculature through adhesion molecules, including LFA-1/ICAM-1 and VLA-4/VCAM-1 interactions, allowing firm adhesion to endothelial cells. (4) Extravasation: CD8⁺ T cells cross the endothelial barrier through diapedesis to access the tumor tissue. (5) Intratumoral migration: Once inside the tumor, CD8⁺ T cells navigate the ECM and tumor stroma through amoeboid migration to reach malignant cells and establish productive immune interactions. Failure at any of these sequential steps can contribute to immune exclusion and impaired antitumor immunity

The accumulation of CD8⁺ T cells within tumors is a coordinated multistep process encompassing lymphoid priming, systemic trafficking, tissue entry, and early adaptation within peripheral tissues. Each stage is governed by distinct molecular mechanisms and represents a potential point of failure that can compromise anti-tumor immunity. Viewing CD8⁺ T-cell recruitment as this continuum is essential for understanding patterns of tumor immune infiltration and for developing rational therapeutic strategies (Fig. 1).

### Priming and activation of tumor-specific CD8⁺ T cells

The priming and activation of tumor-specific CD8⁺ T cells constitute the first essential step in generating an effective anti-tumor immune response. This process is initiated by dendritic cells (DCs), which capture tumor antigens and present them on MHC-I molecules to naïve CD8⁺ T cells, primarily in tumor-draining lymph nodes (TDLNs) [15]. Effective activation of these lymphocytes relies on the integration of three signals: antigen recognition via the TCR (signal 1), co-stimulatory signals such as the interaction between CD80/CD86 and CD28 (signal 2), and cytokine-mediated signals that guide T cell differentiation and functional programming (signal 3). This activation leads to clonal expansion and differentiation of CD8⁺ T cells into cytotoxic effector cells and memory cells capable of migrating to the tumor site [16]. In addition to TDLNs, TLSs can also support local antigen presentation and T-cell priming within or adjacent to the TME. In non-small cell lung cancer (NSCLC), mature TLS-associated DCs directly prime tumor-specific CD8⁺ T cells, promoting their local expansion and differentiation independently of secondary lymphoid organs [17].

### Systemic trafficking toward tumors

#### *CD8*^+^*T cell chemotaxis toward the tumor*

CD8⁺ T-cell recruitment to tumors is primarily driven by chemokine gradients. The main receptors involved include CCR2, CCR5, CCR6, CXCR3, CXCR6, and CX3CR1, each recognizing specific ligands and contributing to T-cell migration toward tumors.

CXCR3 is highly expressed on activated effector T cells and binds CXCL9, CXCL10, and CXCL11, chemokines induced by IFN-γ and associated with "hot" TMEs. Beyond promoting tumor homing, CXCR3 directs T cells to inflammatory intratumoral niches enriched in DCs. Its expression correlates with improved prognosis and better responses to ICIs [18–23]. Although CXCL11 also binds CXCR3, it preferentially activates the CXCR3-B and CXCR3-alt isoforms, triggering immunoregulatory pathways associated with regulatory T cell (Treg) recruitment, PD-L1 upregulation, and increased production of immunosuppressive cytokines [24, 25]. CCR5 also plays an important role in the recruitment of CD8⁺ T cells by recognizing the ligands CCL3, CCL4, and CCL5. This receptor contributes to the amplification of anti-tumor immune responses by promoting the accumulation of effector T cells in the TME in murine models [26–28]. CCR5 expression on CD8⁺ T cells is induced by cytokines such as IL-2 and IL-12 [29, 30]. It is also regulated by interactions with high endothelial venules (HEVs) and can influence the generation and long-term maintenance of CD8⁺ T cell memory in mice [31]. CCR5 ligands are produced by many cell types, including T lymphocytes, monocytes, natural killer (NK) cells, epithelial cells, and fibroblasts, and CCL3 and CCL4 secretion by DC–CD4^+^ T cell complexes attracts naïve CD8⁺ T cells in mice [32]. CCR2 and CCR6 have more context-dependent functions. The CCR2–CCL2 axis recruits CD8⁺ T cells but also attracts immunosuppressive myeloid cells [33]. CCR6, through CCL20, promotes trafficking to inflamed tissues and tumor niches, and loss of CCR6 expression is associated with melanoma metastasis [34]. CX3CR1, the receptor for CX3CL1 (fractalkine), supports adhesion, vascular patrolling, survival, and tumor infiltration. Approximately 10% of circulating CD8⁺ T cells express CX3CR1 [35]. Its expression is associated with improved responses to anti-PD-1 therapy and chemo-immunotherapy in NSCLC, as well as better prognosis in melanoma [36, 37]. Furthermore, high expression of CX3CL1 by tumor cells promotes CD8⁺ T cell recruitment [38]. CX3CR1 also marks highly differentiated cytotoxic T cells, although some studies report reduced cytokine production despite low immune checkpoint expression [36, 39, 40]. Finally, CXCR6 is increasingly recognized as a marker of tissue-resident or semi-resident T lymphocytes [41]. Its main ligand, CXCL16, is frequently expressed by tumor and stromal cells and particularly contributes to the retention and stable localization of CD8⁺ T cells within the tumor tissue, thereby promoting durable local anti-tumor immunity [42, 43].

#### Interaction with the vascular endothelium

Beyond chemotactic guidance, effective CD8⁺ T-cell entry into tumors requires interaction with the vascular endothelium, a critical checkpoint that enables the transition from blood circulation to tissue infiltration. Activated CD8⁺ T cells express adhesion molecules of the integrin family, mainly LFA-1 and VLA-4. These integrins play a central role in lymphocyte recognition and anchoring to the endothelial surface. Their functional activation is regulated by so-called “inside-out” signaling, often triggered by chemokine receptor activation, thereby coupling chemotactic sensing with physical adhesion. On the tumor endothelium side, the main ligands for these integrins are ICAM-1 and VCAM-1. The interactions LFA-1–ICAM-1 and VLA-4–VCAM-1 ensure firm adhesion of T lymphocytes, resistant to the shear forces of blood flow. This step is essential for complete arrest of the cells, a prerequisite and indispensable condition for diapedesis [44, 45].

### Tumor vascular entry

Once firmly adhered to the endothelium, CD8⁺ T cells cross the endothelial barrier by extravasation, a process requiring coordinated remodeling of both the endothelium and the T-cell cytoskeleton. This step depends on endothelial activation. Indeed, under the influence of pro-inflammatory cytokines such as IFN-γ and TNF-α, produced by T cells and NK cells, endothelial cells upregulate ICAM-1 and VCAM-1 and acquire an activated phenotype characterized by increased vascular permeability, thereby facilitating T-cell transmigration [46]. However, tumor-associated vasculature frequently exhibits structural and functional abnormalities, including disorganized vessels, altered blood flow, abnormal endothelial junctions, and impaired pericyte coverage [47, 48]. These abnormalities can compromise leukocyte adhesion and transmigration and thus constitute an important barrier to the effective entry of CD8^+^ T cells into the tumor parenchyma.

Moreover, tumor-associated endothelial cells may develop immunosuppressive properties that hinder the recruitment and entry of effector T cells. For instance, increased expression of the endothelin B receptor can reduce T-cell adhesion to the tumor vasculature, while tumor-derived mediators, including VEGF-A, IL-10, and PGE2, promotes FasL expression on endothelial cells. This endothelial barrier can selectively impair the access of effector CD8^+^ T cells to the tumor tissue [49, 50]. Thus, tumor vasculature not only provides a physical route for T-cell entry but can also actively regulate which lymphocyte populations gain access to the tumor.

These mechanisms provide a rationale for targeting the tumor vasculature to improve T-cell infiltration. Pro-angiogenic factors, particularly VEGF and angiopoietin-2, contribute to vascular abnormalities and immunosuppression, whereas anti-angiogenic treatments can partially restore vascular function [51]. More recently, tumor-associated vasculature has been recognized as a central regulator of T-cell homing, positioning, and function, supporting the development of therapeutic strategies that combine vascular normalization with approaches aimed at enhancing T-cell activation and trafficking [47, 52].

### Intratumoral migration and spatial positioning

Once extravasated, CD8⁺ T lymphocytes must migrate through the TME using an amoeboid mode of migration driven by dynamic actin remodeling, enabling movement through the dense tumor stroma [53]. This mobility depends on tightly regulated actin dynamics. Through this migratory mode, CD8⁺ T cells are able to navigate dense and heterogeneous tissues, including the tumor stroma.

A determinant of this migration is the architecture of the ECM [54]. Many tumors exhibit a dense and disorganized ECM enriched in collagen and fibronectin, whose stiffness increases with tumor progression [55]. This ECM densification reduces porosity and constitutes a physical barrier that limits the mobility of CD8⁺ T lymphocytes.

The CXCR3 axis also plays a key role in the spatial organization of CD8⁺ T cells and reduced CXCR3 axis activity can contribute to a peripheral distribution of T cells and diminished functional efficacy [56–59]. The localization and spatial organization of CD8⁺ T cells influence the likelihood of antigen recognition and, consequently, tumor sensitivity to immunotherapies [60, 61]. Spatial organization is further supported by CXCL13, produced by follicular helper T (Tfh) cells and TGF-β-stimulated stromal cells. CXCL13 promotes the TLS by recruiting lymphocytes and is associated with improved responses to anti-PD-1 therapy [62].

Finally, the CCL20–CCR6 axis, which is less extensively studied, is involved in the organization of local immune responses. CCL20 is produced by lymphocytes and several tissues, including lymph nodes, the liver, and the thymus, and appears to play a role in recruiting immune cells to inflamed tissues and certain specialized tumor niches [63].

### Retention, persistence and residency

Beyond recruitment and migration, the retention and persistence of CD8⁺ T cells within tumors are critical for durable anti-tumor immunity. Effective tumor control depends not only on T-cell infiltration but also on their ability to remain in the tumor, maintain cytotoxic function, and provide long-term immune surveillance.

A key population is tissue-resident memory T cells (TRM), which stably reside within peripheral tissues, including tumors, without recirculating [64]. They express residency-associated markers such as CD69 and CD103, which respectively limit tissue egress and promote stable retention [65–67]. In many cancers, TRM-like CD8⁺ T cells are associated with improved prognosis and enhanced responses to immunotherapy, highlighting their central role in local immune control [68].

T-cell retention is regulated by the CXCR6–CXCL16 axis, which contributes to the maintenance of T cells within tissues [41, 69]. CXCL16, expressed by tumor, endothelial, and stromal cells, exists in both membrane-bound and soluble forms, thereby providing both adhesion-like retention signals and chemotactic guidance [70]. Expression of CXCR6 on CD8⁺ T lymphocytes is associated with a tissue-resident phenotype [42]. These CXCR6⁺ cells often retain durable cytotoxic functionality, thereby contributing to sustained local anti-tumor immunity [71].

Prolonged interactions with antigen presenting cells (APCs), particularly DCs located within intratumoral immune niches or TLS, provide chronic low-intensity antigenic stimulation that promotes the maintenance of CD8⁺ T lymphocytes at the tumor site. However, chronic antigen exposure can also promote T-cell exhaustion, reducing proliferation and cytokine production [72].

Finally, metabolic constraints within the TME—including hypoxia, nutrient deprivation, and the accumulation of adenosine and lactate—further impair CD8⁺ T-cell survival and function [73].

## Barriers limiting CD8⁺ T-cell infiltration and spatial organization

The effective accumulation and organization of CD8⁺ T cells within tumors require overcoming multiple barriers within the tumor microenvironment. Following their recruitment and entry through the tumor vasculature, T cells must navigate the extracellular matrix and stromal compartment, respond to local chemotactic signals, and overcome immunosuppressive signals that may limit their positioning, persistence, and function. These interconnected barriers determine not only whether CD8⁺ T cells reach the tumor, but also where they localize within it and their ability to establish an effective antitumor response.

**Fig. 2:**
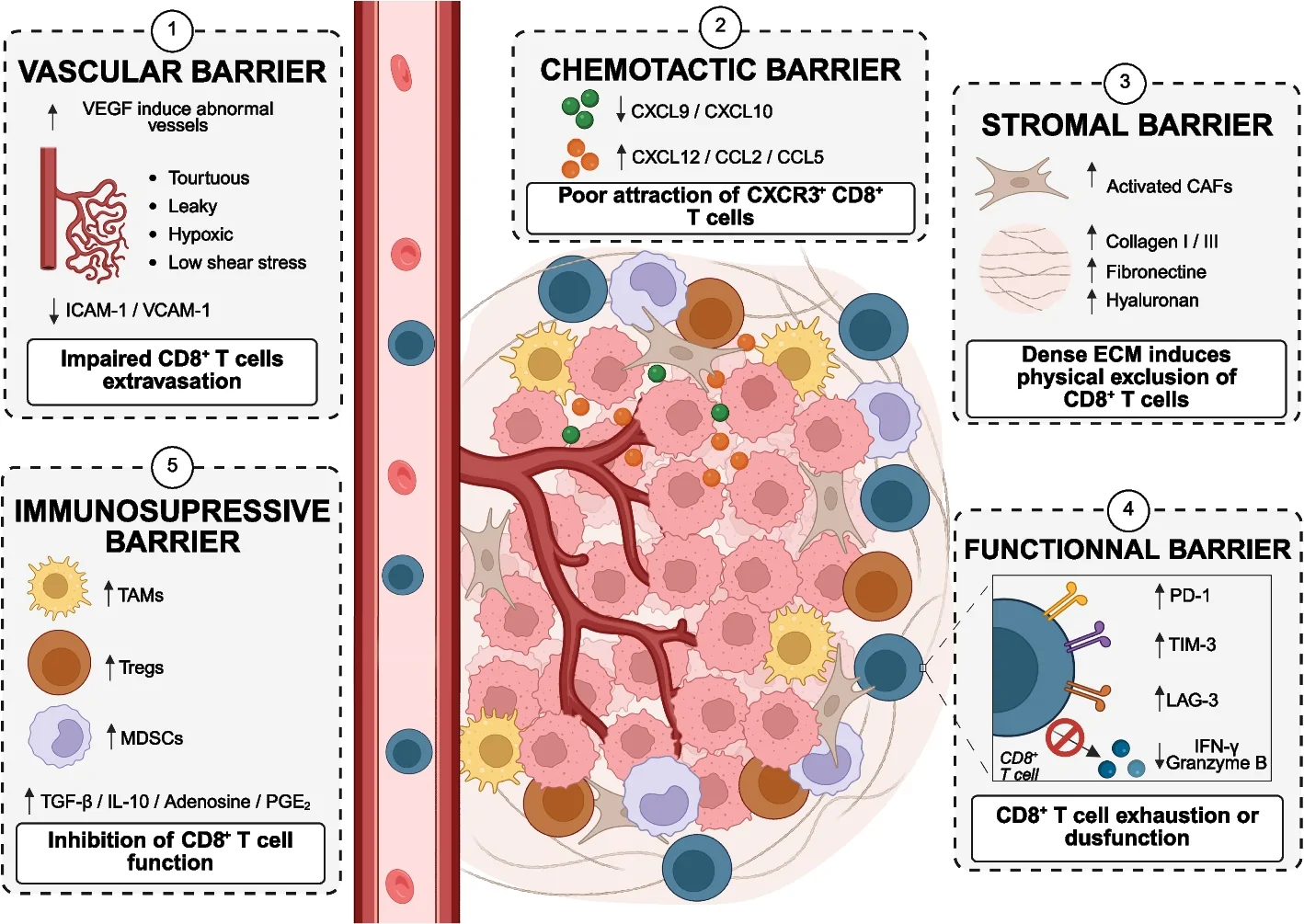
Multilevel barriers limiting CD8⁺ T-cell infiltration and antitumor function within the tumor microenvironment. Tumor immune exclusion results from the coordinated action of vascular, chemotactic, stromal, immunosuppressive, and functional barriers that prevent effective CD8⁺ T-cell accumulation and activity. (1) Vascular barrier: Tumor-associated angiogenesis driven by VEGF generates abnormal, tortuous, and hypoxic vessels with reduced endothelial activation and decreased expression of adhesion molecules (ICAM-1 and VCAM-1), thereby impairing CD8⁺ T-cell adhesion and extravasation. (2) Chemotactic barrier: Altered chemokine production within the TME, characterized by reduced CXCL9/CXCL10 gradients and increased immunosuppressive chemokines such as CXCL12, CCL2, and CCL5, limits the recruitment and spatial guidance of CXCR3⁺ CD8⁺ T cells. (3) Stromal barrier: CAF-mediated ECM remodeling, including increased collagen, fibronectin, and hyaluronan deposition, creates a dense physical barrier that restricts lymphocyte migration toward tumor nests. (4) Functional barrier: Chronic antigen stimulation and suppressive signals promote CD8⁺ T-cell dysfunction and exhaustion, characterized by increased expression of inhibitory receptors (PD-1, TIM-3, LAG-3) and reduced effector functions. (5) Immunosuppressive barrier: TAMs, Tregs, MDSCs, and soluble mediators including TGF-β, IL-10, adenosine, and PGE₂ contribute to impaired CD8⁺ T-cell recruitment and cytotoxic activity

The barriers described below are summarized in Fig. 2.

### Trafficking failure

#### Loss of directional signals

A first level of dysfunction involves the production and spatial organization of chemokines within the TME. Under physiological conditions, chemokines such as CXCL9, CXCL10, and CCL5 establish gradients that guide effector CD8⁺ T cells. In tumors, these gradients are often reduced, heterogeneous, or spatially mislocalized, impairing T-cell recruitment or directing lymphocytes toward stromal or vascular regions rather than toward tumor cells.

Chemokines are produced by tumor, endothelial, and immune cells, including CD103⁺ DCs, tumor-associated macrophages (TAMs), and myeloid-derived suppressor cells (MDSCs). CXCL9 and CXCL10 produced by CD103⁺ DCs are major drivers of CD8⁺ T-cell infiltration [74], and tumor-derived CXCL10 also contributes to immune-mediated tumor dormancy [75]. CXCL10 expression is induced by interferons and activation of the cGAS–STING pathway [76, 77] but can be disrupted by oncogenic events [78]. In melanoma models, constitutive activation of β-catenin impairs the recruitment of CD103⁺ DC and diminishes CXCL9 and CXCL10 production, thereby limiting CD8⁺ T-cell accumulation within tumors and contributing to resistance to immunotherapy [79]. This study provided one of the first mechanistic links between an intrinsic oncogenic pathway and defective chemokine production driving immune exclusion. Similarly, TP53 mutations and epigenetic regulation of CXCL10 influence immunotherapy responses in prostate cancer [80]. Chemokine expression can also be epigenetically repressed in ovarian cancer. Peng and colleagues showed EZH2 and DNMT1-mediated epigenetic repression decreases CXCL9 and CXCL10 expression, thereby reducing the recruitment and infiltration of CD8^+^ T cells into the tumor [81]. Tumor hypoxia also contributes to chemokine dysregulation. Activation of HIF-1α alters the expression of multiple chemokines and often favors recruitment of suppressive myeloid populations rather than effector T cells [82]. In parallel, immunosuppressive cytokines such as TGF-β can indirectly reduce the production of inflammatory chemokines, particularly CXCL9 and CXCL10, thereby reinforcing the immune-excluded phenotype characteristic of many “cold” tumors [83].

Conversely, some chemokines are overexpressed and contribute to the establishment of an immunosuppressive microenvironment. Excessive production of CCL2 by tumor or stromal cells promotes recruitment of inflammatory monocytes and MDSCs, which subsequently suppress T-cell responses [84]. Studies across multiple tumor models have shown that blockade of the CCL2 axis reduces accumulation of these suppressive populations and enhances antitumor immunity [85] although CCL2 can also attract cytotoxic T cells in specific settings such as three-dimensional melanoma models [86].

Finally, the CXCL12–CXCR4 axis represents a major mechanism of T-cell exclusion. Feig and colleagues demonstrated in pancreatic ductal adenocarcinoma (PDAC) that cancer-associated fibroblasts (CAFs) produce large amounts of CXCL12, establishing a stromal chemokine barrier T-cell access to the tumor parenchyma. Pharmacological inhibition of CXCR4 disrupted this barrier and promoted T-cell infiltration into tumors [87, 88]. This mechanism has recently been confirmed by spatial transcriptomics and scRNA-seq experiments in mice [89].

#### Loss of cellular responsiveness

A second, less explored level of dysfunction concerns the expression and function of chemokine receptors on CD8⁺ T cells. Although their regulation is well characterized under physiological conditions, little is known about how the TME alters their expression and, consequently, CD8⁺ T-cell recruitment and spatial organization. Reduced receptor expression, impaired function, or defective downstream signaling are all likely to compromise chemotaxis despite the presence of chemokines in the TME, but direct evidence remains limited.

CXCR3 expression is regulated by several pathways. It is induced by TCR signaling, although the transcription factor KLF2 negatively regulates its expression depending on the intensity and duration of TCR stimulation [90]. Type I interferons, IFN-γ, IL-12, and the transcription factor T-bet all promote CXCR3 expression [91, 92], suggesting that a type I interferon–rich environment may promote efficient recruitment and spatial organization of CD8⁺ T cells. Notably, CXCR3 induction requires both transient TCR signaling and IFN-γ, suggesting that chronic stimulation in tumors may impair its expression [93]. Therefore, chronic stimulation, as observed in the tumor context, may be detrimental to CXCR3 expression. Finally, TGF-β suppresses CXCR3 expression through a SMAD2/3-dependent pathway [94].

A recent review by Mempel and Malehmir, based on RNA-seq data from Prokhnevska et al., showed that chemokine receptor expression changes dynamically during CD8⁺ T-cell differentiation, from naïve cells in TDLNs to effector and terminally differentiated cells within the TME [95, 96]. With the advent of spatial transcriptomics, it should become possible to determine how intratumoral localization influences chemokine receptor expression and how the TME shapes the spatial organization of anti-tumor immunity.

### Stromal and physical exclusion barriers

CD8⁺ T-cell infiltration depends not only on chemotactic cues but also on overcoming physical and structural barriers that promote immune exclusion.

A major barrier is the abnormal tumor vasculature, driven largely by VEGF. Tumor vessels are disorganized, poorly perfused, and dysfunctional, with reduced expression of endothelial adhesion molecules, thereby limiting CD8⁺ T-cell adhesion, extravasation, and entry into tumors [46]. A study using intravital microscopy in a murine model of pancreatic neuroendocrine tumors demonstrated reduced leukocyte adhesion associated with malignancy, concomitant with the emergence of structural abnormalities such as tumor vessel dilation [97]. Interactions between cytotoxic effector CD8⁺ T lymphocytes and the tumor vascular interface are reduced in several murine models of solid cancers, including melanoma, CRC, and pancreatic neuroendocrine tumors, compared with control tissues [98].

The ECM represents a second major physical constraint. Tumors develop a dense, fibrotic, ECM enriched in collagen and fibronectin. Increased ECM stiffness mechanically limits the migration of CD8⁺ T lymphocytes within tumor regions [99]. The main protein components include collagen, elastin, fibronectin, laminin, and proteoglycans, with collagen being the most abundant protein in the ECM. Collagen forms fibers that provide tissues with mechanical strength and rigidity [100, 101]. Beyond collagen other ECM components also strongly modulate ECM stiffness [102]. Cancer cells, mainly through CAFs, remodel the ECM to create a microenvironment that favors tumor growth while mechanically restricting CD8⁺ T-cell migration [103, 104].

CAFs also reinforce immune exclusion by creating CXCL12-rich stromal niches that trap CXCR4-expressing T cells at the tumor periphery. In PDAC, FAP⁺ CAFs produce CXCL12, thereby retaining CD8^+^ T cells within the stromal compartment and limiting their access to tumor cells. Pharmacological inhibition of CXCR4 with AMD3100 (Plerixafor) redistributed T cells into tumor nests, halted PDAC growth, and dramatically enhanced anti-PD-L1 therapy, nearly eliminating tumor cells after six days of combined treatment [87]. Beyond PDAC, CAFs restrict CD8⁺ T-cell infiltration in several cancers. In breast cancer, spatial transcriptomics identified CAF subsets associated with reduced infiltration and dysfunction of TCF1⁺GZMK⁺ CD8⁺ T cells, partly through Gal9-mediated suppression [105]. Tenascin-C further contributes to immune exclusion by binding CXCL12 and forming a stromal scaffold that retains tumor-infiltrating lymphocytes. In patients, low tenascin-C expression combined with high CD8⁺ tumor-infiltrating lymphocytes density is associated with longer metastasis-free survival [106].

### Immunosuppressive TME constraining CD8⁺ T-cell recruitment

Several immunosuppressive components of the TME can affect CD8^+^ T cell recruitment.

TGF-β is a cytokine produced by cancer cells, CAFs, and certain anti-inflammatory immune cells. It plays multiple and complex roles during tumorigenesis. TGF-β exerts immunosuppressive functions but can also reduce CD8⁺ T-cell infiltration in tumors by inhibiting CXCR3 expression [94]. Certain CAF subtypes (Endo180⁺ CAFs) have also been described as inhibiting CD8⁺ T-cell infiltration through high levels of TGF-β production [105].

TAMs can polarize toward an M2 phenotype with pro-tumoral and anti-inflammatory functions. Differentiation inhibitors (ID1) expressed by TAMs are responsible for T-cell exclusion from tumors [107]. In parallel, IL-15Rα⁺ TAMs reduce CD8⁺ T-cell recruitment in breast cancer by decreasing CX3CL1 levels [108].

Adenosine is a major immunosuppressive metabolite, contributing to the establishment of molecular and cellular barriers that limit CD8⁺ T-cell function and infiltration. It accumulates primarily in hypoxic and highly glycolytic tumor regions [109], where extracellular ATP released by stressed or dying cells is rapidly converted into adenosine through the sequential enzymatic activity of CD39 (ENTPD1) and CD73 (NT5E), expressed by tumor, stromal, endothelial, and regulatory immune cells. At the molecular level, adenosine acts mainly through A2A and A2B receptors expressed on CD8⁺ T cells [110]. Activation of these Gs-coupled receptors increases intracellular cAMP, inhibits TCR signaling, reduces cytotoxic granule release, and decreases effector cytokine production such as IFN-γ and TNF-α [111]. Adenosine also disrupts the migration processes required for CD8⁺ T-cell infiltration [112].

PGE_2_ is a lipid mediator derived from arachidonic acid [113]. In the TME, PGE_2_ is frequently overproduced by tumor cells, CAFs, and myeloid populations [114–118]. It plays a central role in establishing molecular and cellular barriers that limit CD8⁺ T-cell infiltration and function. At the molecular level, PGE_2_ signals through four G protein–coupled receptors (EP1–EP4 receptors) [119]. Their activation triggers cAMP-dependent pathways [120]. PGE_2_ also disrupts chemokine networks required for T-cell recruitment by decreasing the production of key chemokines such as CXCL9 and CXCL10 [121].

## Therapeutic strategies to enhance CD8⁺ T-cell tumor infiltration

**Fig. 3:**
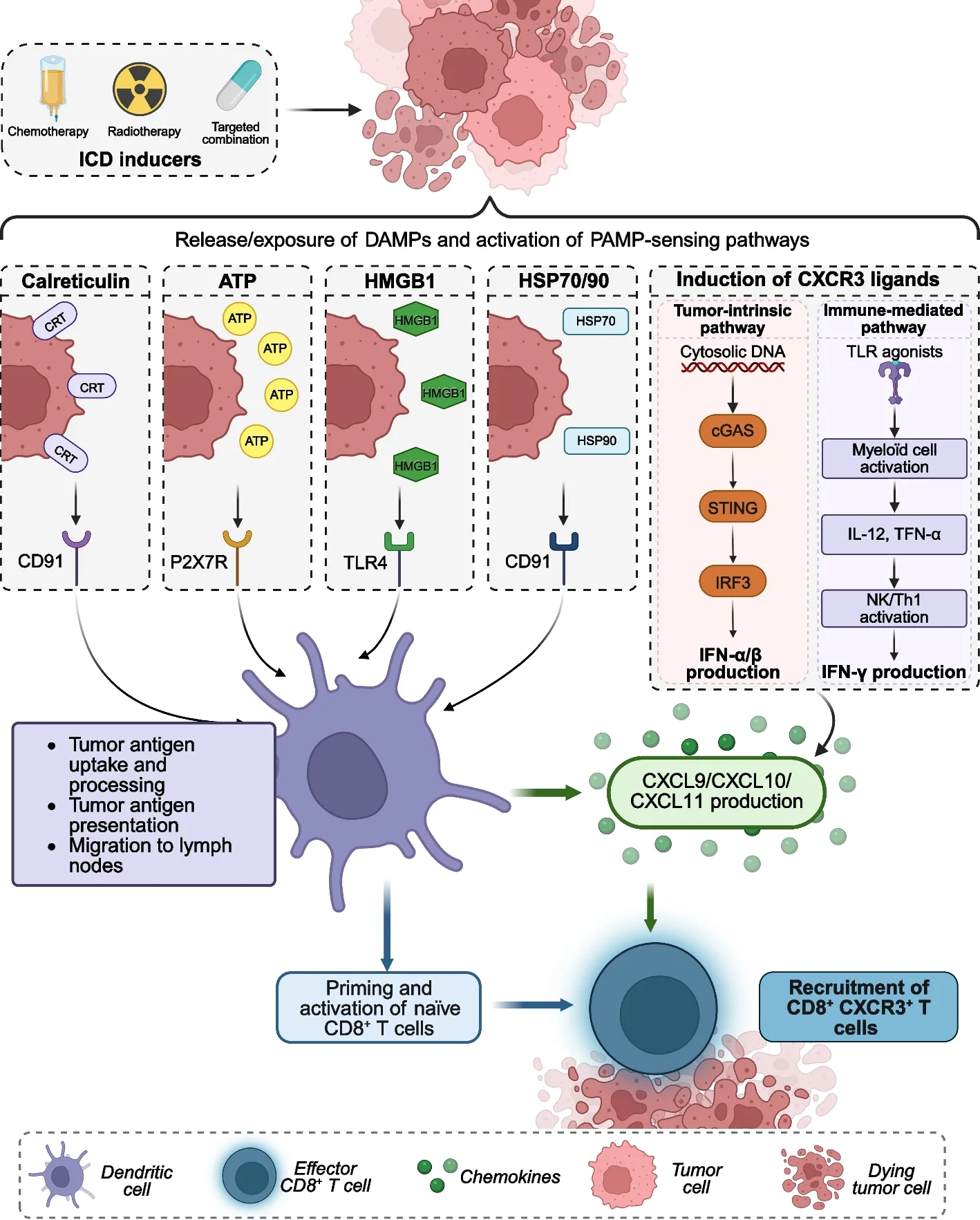
Therapeutic strategies to target major barriers limiting CD8⁺ T-cell infiltration and function in the tumor microenvironment. Key mechanisms include abnormal tumor vasculature, stromal barriers, immunosuppressive signaling, reduced CXCR3 ligand expression, chemokine-mediated T-cell exclusion, immune checkpoint-mediated T-cell dysfunction, and the absence or paucity of TLS

Overcoming the barriers that limit CD8⁺ T-cell infiltration requires therapeutic strategies targeting different and interconnected steps of the antitumor immune response. These approaches focus on restoring initial T-cell activation, modulating chemokine networks, tumor vasculature, stromal barriers, and immunosuppressive pathways. Targeting these mechanisms may have complementary effects not only on CD8⁺ T-cell recruitment and entry into the tumor, but also on their spatial organization, persistence, and effector function, thereby providing a rationale for the development of rational combination therapies. The different strategies discussed below are summarized in Fig. 3.

### Restoring immune priming and antigen presentation

Efficient CD8⁺ T cell accumulation within tumors depends not only on the mechanisms governing tissue entry but also the generation of an adequate tumor-specific T-cell repertoire. Defects occurring during tumor antigen release, uptake, processing, or presentation can restrict the magnitude and quality of the ensuing CD8 + T-cell response. Therapeutic interventions that restore these upstream events may therefore increase the pool of tumor-reactive T cells available for subsequent trafficking and tumor infiltration.

**Fig. 4:**
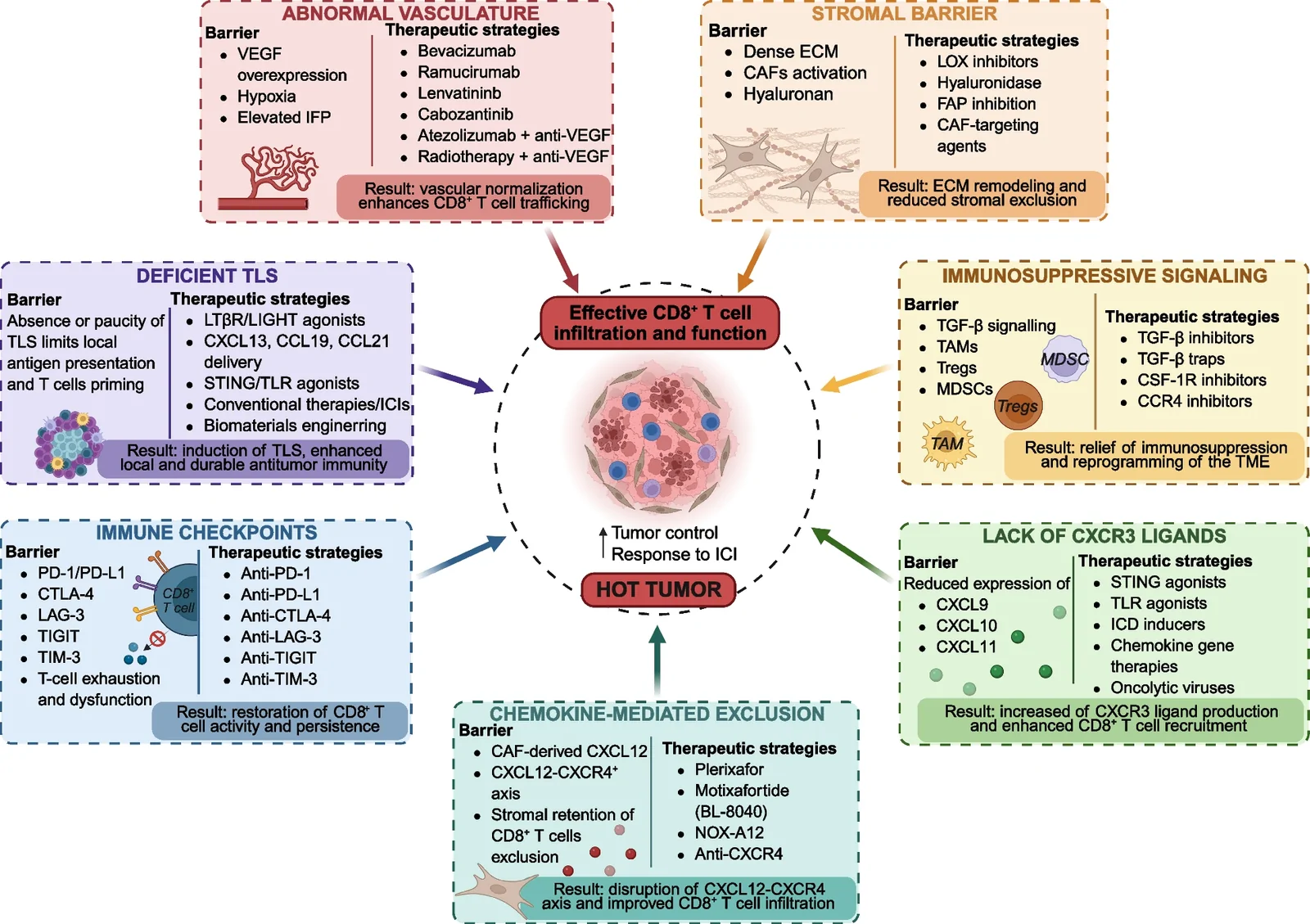
Mechanisms promoting CXCR3-dependent recruitment of CD8⁺ T cells into tumors. Therapeutic interventions, including chemotherapy, radiotherapy, and targeted combinations, induce the release or exposure of DAMPs, including calreticulin (CRT), ATP, HMGB1, and HSP70/90. These molecules activate DCs through CD91, P2X7R, and TLR4, promoting tumor antigen uptake, processing, migration to TDLNs, and priming of naïve CD8⁺ T cells. In parallel, activation of the tumor-intrinsic cGAS–STING pathway and immune-mediated inflammatory signaling induces type I interferons and IFN-γ, leading to the production of the CXCR3 ligands CXCL9, CXCL10, and CXCL11. The resulting chemokine gradient promotes the recruitment of CXCR3⁺ effector CD8⁺ T cells into the tumor bed, thereby enhancing antitumor immunity

One therapeutic strategy aimed at improving immune priming consists of increasing the availability and immunogenicity of tumor antigens. Certain conventional therapies can induce a form of cell death known as immunogenic cell death (ICD), leading to the release of tumor antigens as well as danger-associated molecular patterns (DAMPs). These include calreticulin, ATP, and HMGB1 [122–124]. The release of these DAMPs is associated with the exposure or secretion of HSP70 and HSP90 at the cell surface, which correlates with DC activation [125]. Activated DCs then migrate to lymph nodes, where they prime naïve CD8⁺ T cells. During ICD, pathogen-associated molecular pattern (PAMPs) sensors are essential for the production of type I interferons and CXCL10 [126] (Fig. 4). However, the ability of conventional chemotherapy to elicit a fully immunogenic form of cell death is variable, which has prompted efforts to combine cytotoxic agents with treatments capable of amplifying the associated immune response. For example, it has recently been reported that Cisplatin combined with Tyrosine Kinase Inhibitors (TKIs) improves ICD, enhancing CD8⁺ T-cell infiltration and responses to immunotherapies [127].

A second approach relies on enhancing DC functions. DCs play a central role in CD8⁺ T-cell priming. However, in many tumors, DCs are either excluded from the TME, functionally suppressed, or skewed toward tolerogenic phenotypes. Several therapeutic interventions have been explored to restore DC activity, including FLT3L, CD40 agonism, and pharmacological activation of the STING pathway, with the potential to promote DC maturation, typie I interferon responses, and antigen cross-presentation. In murine model, combined FLT3L and CD40 agonist treatment expanded the cDC1 compartment and enhanced CD8^+^ T-cell activation in tumor-draining lymph nodes, resulting in increased T-cell trafficking to tumors and reduced tumor growth [128]. The cGAS-STING pathway functions as a DNA-sensing system that responds to both microbial and endogenous DNA. Following DNA detection, cGAS catalyzes the production of cGAMP, which subsequently activates STING and triggers downstream signaling leading to type-I IFN production [129, 130]. Through this signaling cascade, STING activation can engage both innate and adaptive immune compartments, including DCs, NK cells, macrophages, and T lymphocytes. Most evidence supporting STING agonists currently derives from preclinical studies. Clinical translation has yielded mixed results. Intratumoral injection of cGAMP has been reported to induce CD8⁺ T-cell infiltration through STING pathway activation in B16F10 and MC38 models [131, 132]. In glioblastoma, intratumoral injection of a hyaluronic acid–STING agonist conjugate improved CD8⁺ T-cell infiltration by reprogramming the TME [133]. DMXAA has been identified as another STING agonist. In murine tumor models, DMXAA showed promising effects by reducing fibrosarcoma growth and increasing the number of tumor-specific T cells [134]. In a phase II clinical trial, a combination therapy of DMXAA with Carboplatin and Paclitaxel demonstrated superior efficacy in advanced NSCLC compared with chemotherapy alone [135]. However, these favorable results were not reproduced in a subsequent phase III clinical trial. Finally, intratumoral injection of DCs expressing CCL21 increased CD8⁺ T-cell infiltration and enhanced antitumor responses in a phase I/II clinical trial in NSCLC [136]. Despite promising antitumor activity in murine models, the clinical translation of STING agonists has remained challenging. The failure of DMXAA in phase III clinical development highlights the limitations of extrapolating finding from mouse models to humans, particularly given differences between murine and human STING signaling in ligand recognition and downstream responses. Clinical development is also constrained by treatment-related inflammatory toxicity and the short half-life of some compounds, while intratumoral administration limits their use to accessible lesions. Furthermore, the consequences of STING activation may vary according to tumor type and the functional status of the pathway in tumor and stromal cells.

**Fig. 5:**
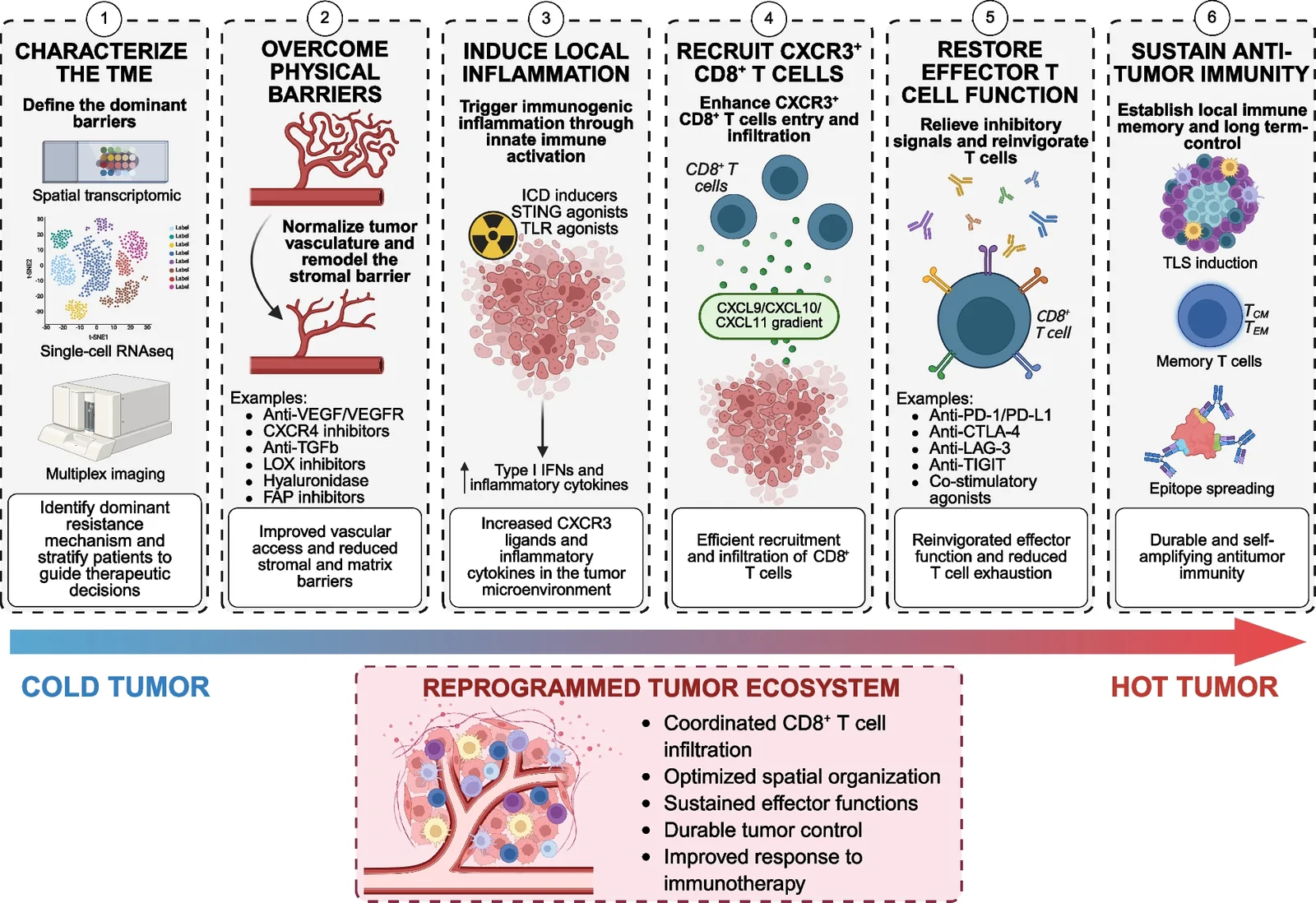
Schematic representation of the stepwise development and maturation of TLS. TLS formation progresses from early lymphoid aggregates, characterized by the production of homeostatic chemokines (CXCL13, CCL19, and CCL21), the development of HEVs, and the recruitment of DCs and lymphocytes, to immature TLS with distinct b-cell follicles and t-cell zones, and finally to mature TLS containing germinal centers with proliferating b cells, Tfh cells, FDCs, and active antigen presentation

The induction of TLS represents another key mechanism to enhance local immune priming. Given the association between TLS and favorable responses to immunotherapy, several studies have explored different strategies to induce TLS formation in vivo (Fig. 5).

Local TLS induction in murine models has been achieved by stimulating the expression of cytokines and chemokines such as LT-α [137], LIGHT [138], CCL21, CCL19, and CXCL12 [139]. CXCL13 has also been investigated, although results remain context-dependent and sometimes contradictory [140, 141]. Chemotherapy has also emerged as a potential modulator of TLS development and maturation. In a recent study of high-grade serous ovarian cancer bearing patients, platinum- and taxane-based neoadjuvant chemotherapy increased antigen release and promoted the generation of germinal center-like TLS at metastatic sites [142]. Radiotherapy has also been shown to promote TLS formation and enhance TLS maturation in murine models of lung adenocarcinoma [143]. Radiotherapy may promote TLS development through a distinct mechanism, involving local DNA damage and the release of danger-associated signals (double-stranded DNA and DAMPs), which activate the cGAS–STING pathway and support chemokine and vascular remodeling programs. In a complementary approach, an exosomal system derived from irradiated tumors in mice prolonged STING activation and promoted HEV remodeling, thereby increasing TLS density [144]. These findings suggest that radiotherapy may act as an immune-priming intervention by activating chemokine and vascular programs that support TLS development. Evidence from several tumor types indicates that ICI treatment can promote TLS development and maturation. In bladder cancer patients, ICIs induce B-cell expansion, Tfh cell activation, and the formation of immature TLS, establishing a link between checkpoint blockade and TLS induction [145, 146]. Mechanistic studies in the MC38 mouse model demonstrated that combined PD-1 and CTLA-4 blockade increases CXCL13 and CCL19 expression, promoting lymphocyte aggregation and TLS assembly [138]. Findings in PDAC mouse model further support this paradigm: KRAS inhibition combined with CTLA-4 blockade remodels the TME and promotes TLS maturation [138]. In Human hepatocellular carcinoma (HCC), neoadjuvant ICIs significantly increase intratumoral TLS density and are associated with higher pathological response rates and prolonged recurrence-free survival [147, 148], as well as, in gastric adenocarcinoma, NSCLC and melanoma [17, 145, 149–151]. Other immunotherapeutic approaches, such as administration of an allogeneic vaccine (GVAX) in PDAC, have induced intratumoral TLS in 85% of patients [152]. By inducing tumor-cell lysis and releasing immunostimilatory signals, oncolytic viruses can create local conditions that favor TLS development. In several mouse models, oncolytic herpesvirus induced upregulation of the CXCL10–CXCR3 axis, recruitment of TCF1⁺ CD8⁺ T cells, and TLS formation [153]. Early clinical data support these findings in infiltrating bladder cancer and melanoma [154, 155]. Intratumoral administration of DCs expressing TBX21, and induction of TNF-α and IL-1β expression by mesenchymal stem cells have also been investigated as strategies to promote TLS formation in mouse tumors [156, 157].

Although TLS induction is increasingly explored as a therapeutic strategy, their presence alone does not necessarily ensure effective anti-tumor immunity. Their functional impact depends on their degree of maturation, cellular composition, vascular organization, and ability to sustain productive local antigen presentation. An important challenge will therefore be to establish biomarkers capable of discriminating functionally active TLS from structures with limited immunological activity.

### Reprogramming chemokine networks and T-cell trafficking

Disrupted chemokine signaling is an important contributor to ineffective T-cell trafficking within tumors. Accordingly, restoring or redirecting chemokine networks has emerged as a potential strategy to improve antitumor immune responses.

The CXCR3-CXCL9/CXCL10 axis is a central determinant of CD8^+^ T-cell recruitment into tumors. However, many tumors exhibit impaired production of these chemokines. Strategies aimed at restoring CXCL9 and CXCL10 within the TME have consequently been explored to reinforce local CD8^+^ T-cell recruitment. Several approaches, including intratumoral chemokine delivery, activation of innate immune pathways, or stimulation of IFN signaling, aim to reconstruct local chemotactic gradients and promote the accumulation of cytotoxic lymphocytes within tumor lesions (Fig. 4). Some chemotherapies can induce a form of ICD responsible for generating such gradients. However, most clinically used chemotherapies induce only a subset of the signals required for complete ICD. Consequently, therapeutic combinations are being explored. For example, it has been demonstrated that lung tumors that fail to respond to Pemetrexed plus Cisplatin combined with immunotherapy display impaired CXCL10 production, which can be restored by MEK inhibitors [127]. Similarly, Olaparib has been shown to enhance radiosensitivity in HCC. This increases DNA double-strand breaks, activates the cGAS–STING pathway, induces type I interferon production, and promotes secretion of CXCL9, CXCL10, and CXCL11 [158, 159] (Fig. 4). Chemotherapy can also induce CXCL10 production independently of ICD. In mouse models of lung cancer, Cisplatin was shown to induce CCL20 and IL-1β production at the tumor site, promoting recruitment of innate lymphoid cells type 3 (ILC3) that subsequently produce CXCL10 [160]. However, several studies indicate that some Tregs can also express CXCR3 and migrate toward CXCL10-rich microenvironments, where they contribute to local immunosuppression. Therefore, CXCL10 may exert dual effects depending on the tumor context and the balance between CD8⁺ T cells and Tregs within the TME [161].

An alternative approach is to interfere with chemokine pathways that actively maintain immune exclusion. Among these pathways, CXCL12-CXCR4 signaling has been implicated in the establishment of tumor niches that restrict effector T-cell access. Pharmacological inhibition of CXCR4 has shown, in preclinical models, the ability to disrupt this stromal barrier, improve CD8⁺ T-cell infiltration, and enhance responses to ICIs. The CXCR4 antagonist Mavorixafor was approved by the Food and Drug Administration (FDA) in 2024 [162, 163]. Another CXCR4 inhibitor, POL6326, has also shown promising potential [164]. Although CXCR4 inhibition successfully enhanced T-cell recruitment, clinical efficacy has remained limited, suggesting that increased immune infiltration alone is insufficient when other immunosuppressive circuits remain active. Indeed, blockade of a single chemokine pathway may trigger compensatory recruitment mechanisms through alternative chemokine networks or immunosuppressive myeloid populations, suggesting that CXCR4 inhibition will probably require combination approaches to achieve durable clinical benefit.

An additional emerging strategy consists of modifying the ability of therapeutic T cells to respond to chemokines. Adoptively transferred cells, particularly chimeric antigen receptor (CAR)-T cells, often show limited efficacy in solid tumors due to insufficient trafficking toward tumor sites. Chemokine receptor engineering has therefore been explored as a means of redirecting adoptively transferred T cells toward tumor sites. CAR-T cells have been designed to express receptors capable of sensing chemokines released by tumor cells [165]. But to date, most chemokine receptor-engineered CAR-T strategies remain at the preclinical stage. The CCR2–CCL2 axis is considered one of the major mechanisms involved in intratumoral trafficking of adoptive cell therapies. CAR-T cells expressing CCR2b have demonstrated increased tumor infiltration [166, 167]. Furthermore, engineering T cells to express CXCR2 enables interaction with CXCL1 produced by HCC and pancreatic cancer cells, thereby enhancing T-cell migration toward tumor tissues [14, 168]. CXCR1- and CXCR2-modified CD70-targeting CAR-T cells have also demonstrated increased intratumoral infiltration in an in vivo glioblastoma model [169, 170]. In a preclinical study using orthotopic mouse models of diffuse intrinsic pontine glioma, CAR-T cells engineered to express CXCR3 migrated more efficiently toward CXCR3 ligands in vitro and exhibited enhanced tumor trafficking and improved therapeutic efficacy [171]. Overexpression of CXCR2 and CXCR6 has also been shown to enhance CAR-T-cell infiltration and antitumor activity. Preclinical studies demonstrated that CAR-T cells engineered to express these receptors improved tumor homing and prolonged survival in osteosarcoma models [172]. Genetic overexpression of CXCR4 in CAR-T cells has significantly improved their ability to target tumors in vivo and to infiltrate the bone marrow. In models of both localized and disseminated B-cell lymphoma and multiple myeloma, CXCR4-overexpressing CAR-T cells demonstrated superior therapeutic efficacy compared with second-generation CAR-T cells, even at one-quarter of the infusion dose. More importantly, through enhanced infiltration and accumulation in the bone marrow, CXCR4-engineered CAR-T cells induced long-term immune memory protection in vivo. Based on these results, a first-in-human clinical trial of autologous CXCR4^high^ CD19^+^ CAR-T cells was conducted in patients with relapsed/refractory B-cell lymphoma with positive interim results (NCT04684472) [173]. Finally, one study showed that the migration of effector CAR T cells is improved by CCR4 overexpression; consequently, their anti-lymphoma activity is enhanced in vivo [174]. Overall, these genetic modifications did not alter other aspects of cellular biology.

Improving tumor trafficking alone is nevertheless unlikely to fully overcome the limitations of CAR-T cell therapy in solid tumors. Once within the TME, engineered lymphocytes remain exposed to metabolic stress, persistent antigen stimulation, immunosuppressive cytokines, and heterogeneous antigen expression, all of which contribute to functional exhaustion and limited long-term persistence.

### Tumor vasculature normalization and endothelial activation

Anti-angiogenic therapies are clinically approved for the treatment of several cancers. These treatments aim to inhibit VEGFR-mediated signaling, either through monoclonal antibodies targeting VEGF-A or VEGFR2, or through multi-target TKIs that block multiple receptors involved in angiogenesis. The primary objective of these therapies is to normalize tumor vasculature. This also improves immune cell infiltration, particularly that CD8⁺ T cell infiltration. Indeed, normalized vasculature promotes more efficient immune trafficking, and enhances tumor oxygenation. However, excessive inhibition of tumor angiogenesis can have deleterious effects: it may exacerbate hypoxia by destroying tumor blood vessels, thereby promoting tumor aggressiveness. To address this issue, R.K. Jain introduced the concept of vascular normalization in 2005 [175]. This strategy aims to restore the balance between pro- and anti-angiogenic signals, enabling structural and functional normalization of tumor blood vessels. Importantly, vascular normalization is transient, typically lasting only a few days after the initiation of anti-angiogenic treatment. In murine models, the so-called “normalization window” occurs between 2 and 8 days following anti-VEGFR2 administration [176, 177]. Moreover, low doses of this antibody induce a more uniform and functional vasculature compared with high-dose regimens [178, 179]. In clinical settings, anti-angiogenic agents are generally combined with chemotherapy, radiotherapy, or immunotherapy to exploit this vascular normalization window and improve therapeutic outcomes.

Bevacizumab is the most widely described anti-angiogenic therapy. This humanized monoclonal antibody targets all isoforms of VEGF-A, thereby preventing its binding to its receptors and inhibiting downstream pro-angiogenic signaling pathways. It is used in combination with fluoropyrimidine-based chemotherapy for metastatic CRC and is also indicated for breast cancer, NSCLC, ovarian, fallopian tube and peritoneal cancers, renal cancer, and cervical cancer [180]. Bevacizumab exerts both vascular and immunomodulatory effects, including suppression of neovascularization, regression of abnormal vessels, restoration of pericyte coverage, and progressive normalization of the tumor vasculature [181–183]. It has also been shown to enhance antitumor immunity. In CRC, Bevacizumab reduces circulating Tregs and, when combined with chemotherapy, the effect if even more drastic [184]. In patients with metastatic CRC, Bevacizumab leads to a significant increase in CD4^+^, CD8^+^, CD3^+^, and CD20^+^ lymphocytes after three treatment cycles and, CD3^+^ and CD20^+^ cells remain elevated after six cycles [185]. In melanoma, the combination of Ipilimumab (anti-CTLA-4) with Bevacizumab enhances CD8⁺ T-cell infiltration, likely through increased CXCL10 expression and upregulation of adhesion molecules in the tumor vasculature [186].

Ramucirumab is a fully human monoclonal antibody targeting VEGFR2 [187]. It is indicated for several cancers, including gastric cancer, metastatic CRC, NSCLC, and HCC. It can reverse VEGF-induced inhibition of T-cell proliferation [188]. In patients with metastatic gastric cancer, it increases CD8⁺ T-cell infiltration into tumors while reducing PD-1 expression on these cells. Furthermore, it decreases Treg frequency in the TME, thereby alleviating local immunosuppression [189].

TKIs modulate the TME and enhance antitumor immunity, particularly by promoting CD8⁺ T-cell recruitment through chemokine induction. In melanoma, Sunitinib, a multi-target TKI, increases the expression of CXCL10 and CXCL11, two chemokines essential for CD8⁺ T-cell recruitment [190]. Indeed, VEGF has been shown to inhibit TNF-α–induced secretion of these chemokines, suggesting that TKIs can restore chemokine production by counteracting VEGF-mediated immunosuppression. Finally, in a preclinical MC38 colon carcinoma model, Cabozantinib combined with a therapeutic cancer vaccine significantly increases CD8⁺ T-cell infiltration and enhances their cytotoxic activity [191]. These findings suggest that combining TKIs with immunotherapy may synergistically remodel the TME in favor of effective antitumor immune responses.

However, the benefit of vascular normalization remains highly dependent on treatment timing and dosing. Despite extensive clinical experience with VEGF blockade, improvements in immune infiltration have not uniformly translated into long-term survival benefits, reflecting the adaptive mechanisms that can restore an immunosuppressive tumor environment. Because the normalization window is transient, inappropriate scheduling may instead exacerbate hypoxia and reinforce immune suppression, highlighting the need for optimized treatment sequencing and predictive biomarkers.

### Targeting stromal and ECM barriers

A major determinant of immune exclusion in solid tumors is the presence of physical and architectural barriers imposed by the stromal compartment and the ECM. CAFs play a central role in establishing this barrier. Consequently, therapeutic strategies targeting CAFs have emerged to dismantle this physical barrier and restore immune accessibility.

The composition and physical properties of the ECM can also be modified pharmacologically. Several approaches have been explored to modify the tumor ECM. Losartan, an angiotensin receptor blocker, decreases collagen and hyaluronan production by CAFs in breast and pancreatic cancers, with consequent improvments in vascular perfusion and drug delivery in these malignancies [192, 193]. Similarly, PEGPH20 enzymatically depletes hyaluronan and has been reported to improve the intratumoral delivery of chemotherapeutic agents, including Gemcitabine and Doxorubicin [194, 195]. Tenascin C has been targeted in clinical trials using radiolabeled antibodies and has shown a survival benefit [196]. α-SMA marks the myofibroblastic subpopulation of CAFs. In a spontaneous PDAC mouse model crossed with α-SMA–thymidine kinase transgenic mice, selective depletion of α-SMA⁺ myofibroblasts suppressed tumor angiogenesis. However, this depletion increased tumor hypoxia and induced epithelial–mesenchymal transition features as well as stem-like properties in pancreatic tumor cells. More importantly, targeting α-SMA increased infiltration of Tregs within tumors, ultimately leading to more aggressive tumor progression and reduced animal survival [197].

Because CAFs are more genetically stable than tumor cells, they have also been considered potential targets for immunotherapy. Vaccination strategies directed against the CAF-associated antigen FAP may represent an optimal cancer immunotherapy strategy. In addition, administration of FAP5-DM1, an anti-FAP monoclonal antibody conjugated to a maytansinoid, resulted in sustained tumor growth inhibition and even complete regression without observable toxicity in stromal-rich xenograft models (lung, pancreas, and head and neck cancers) [198]. In a phase I trial in patients with advanced cancers expressing FAP-positive stroma, a humanized antibody (Sibrotuzumab) was well tolerated and showed potential to slow tumor progression [199]. However, a phase II trial in metastatic CRC failed due to limited efficacy [200]. All-Trans Retinoic Acid (ATRA) can also induce CAF quiescence in pancreatic cancer models, reducing desmoplasia and slowing tumor growth [201, 202].

Finally, the antifibrotic drug pirfenidone, originally developed for idiopathic pulmonary fibrosis, has emerged as a promising strategy against tumor desmoplasia. It inhibits TGF-β activity in both tumor and non-tumor cells, leading to reduced deposition of type I fibrillar collagen and hyaluronan in breast tumors, as well as decreased angiogenesis and lung metastasis [203]. It also reduces CAF activation, decreases collagen and periostin deposition, slows tumor growth, and enhances Gemcitabine efficacy in murine pancreatic cancer models [204]. Importantly, CAF populations are highly heterogeneous and include subsets that may restrain tumor progression. Consequently, indiscriminate CAF depletion has produced paradoxical effects in several preclinical models, emphasizing that selective stromal reprogramming is likely to be preferable to complete stromal ablation.

### Combination immunotherapies and rational integration

The recruitment and activity of CD8⁺ T lymphocytes within the TME rely on a multiparametric process that sequentially involves the generation of an immune response, lymphocyte trafficking, tumor infiltration, and the maintenance of an effective cytotoxic function. In many cancers, multiple immune exclusion mechanisms coexist, limiting the efficacy of therapeutic approaches targeting a single step of the immune cycle.

ICIs restore the activity of CD8⁺ T cells that are already present within the tumor; however, their efficacy remains limited in “cold” tumors. Conversely, strategies aimed at increasing CD8⁺ T-cell recruitment can promote their entry into the TME without necessarily overcoming mechanisms of functional exhaustion or local immunosuppression. Similarly, vascular normalization improves the physical access of lymphocytes to the tumor compartment but often requires a pre-existing immune signal to generate an effective immune infiltrate. Therefore, current therapeutic strategies rely on rational combinations capable of converting an immune-excluded tumor into an immunologically permissive environment.

#### Immuno-vascular reprogramming

The combination of chemotherapy inducing ICD and inhibition of the VEGF/VEGFR pathway is based on a strong biological rationale. In metastatic CRC, the phase III trial by Saltz et al. showed that adding Bevacizumab to Oxaliplatin-based chemotherapy (FOLFOX or XELOX) significantly improved progression-free survival (9.4 vs 8.0 months), confirming the clinical feasibility of the chemotherapy–anti-angiogenic combination [205]. More recently, the multicenter trial by Pan et al. evaluated XELOX plus Bevacizumab with or without adoptive cellular immunotherapy using PD-1 blockade–activated DCs in metastatic CRC, illustrating an integrated strategy combining tumor antigen release (Oxaliplatin-induced ICD), vascular normalization (anti-VEGF), and amplification of immune effector responses [206]. In NSCLC, the IMpower150 trial also supported this mechanistic model by showing that the combination of Carboplatin–Paclitaxel–Bevacizumab–Atezolizumab improves overall survival, suggesting synergy between cytotoxic chemotherapy, VEGF inhibition, and immune reactivation via anti–PD-L1 therapy [207]. At the mechanistic level, chemotherapies such as Oxaliplatin and anthracyclines induce ICD, while anti-VEGF therapies contribute to a less immunosuppressive microenvironment by limiting MDSC and Treg infiltration and promoting CD8⁺ T cells. This model is consistent with the recent synthesis by Galluzzi et al., which describes ICD as a central entry point for combinatorial strategies aimed at remodeling the TME [208]. The combination of radiotherapy with VEGF/VEGFR inhibition is another approach under investigation, although clinical evidence remains limited and largely exploratory. In breast cancer brain metastases, a randomized trial published in JAMA Oncology reported that adding Bevacizumab to whole-brain radiotherapy, with or without chemotherapy (Etoposide–Cisplatin), improves intracranial control and brain-specific progression, supporting the relevance of vascular modulation during irradiation [209]. In glioblastoma, several recent meta-analyses evaluated Bevacizumab combined with radiotherapy and Temozolomide, showing improved radiological tumor control but an uncertain overall survival benefit [210, 211]. In parallel, clinical and preclinical studies suggest a radiosensitizing effect of multi-kinase VEGFR inhibitors, particularly Anlotinib, when combined with brain radiotherapy, with improved progression-free survival and local control in brain metastases and high-grade gliomas. In locally advanced cervical cancer, exploratory phase II studies also reported that Anlotinib combined with radiotherapy improves objective response rates and disease control, suggesting a radiosensitizing effect of VEGFR inhibitors [212]. Mechanistically, this synergy is based on the concept of vascular normalization and on the observation that radiotherapy induces tumor antigen release and activation of antigen presentation pathways, thereby promoting CD8⁺ T-cell infiltration. In addition, VEGFR inhibition reduces immunosuppressive populations and restores endothelial adhesion molecule expression such as ICAM-1 and VCAM-1, facilitating lymphocyte trafficking [213]. Together, these mechanisms support the development of triplet strategies combining radiotherapy, anti-VEGF/VEGFR therapy, and anti–PD-1/PD-L1 immunotherapy.

Targeting Toll like receptors (TLRs), particularly TLR7, TLR8, and TLR9, in combination with ICIs (anti–PD-1/PD-L1), represents an immunotherapy strategy aimed at converting “cold” tumors into inflamed tumors through activation of innate immunity. Several recent clinical trials have demonstrated the feasibility and biological interest of this approach. For instance, the phase II trial evaluating the TLR7/8 agonist EIK1001 in combination with Pembrolizumab and chemotherapy in metastatic NSCLC explores a triple-combination strategy designed to enhance immune responses via DC activation and T-cell stimulation (NCT06246110, J Clin Oncol, 2024, [214]). Similarly, interim results from the TransCon TLR7/8 agonist plus Pembrolizumab ± chemotherapy study (NCT05081609, J Clin Oncol, 2024) presented at ASCO 2024, showed systemic immune activation with an overall acceptable safety profile in a combination setting [215]. In the TLR9 agonist class, the VIDUTOLIMOD trial combined with Nivolumab in the neoadjuvant melanoma setting demonstrated a major pathological response rate of approximately 55%, associated with marked activation of plasmacytoid DCs and intratumoral T lymphocytes [216]. Intratumoral delivery approaches such as SD-101 (TLR9 agonist) combined with Pembrolizumab or Nivolumab/Ipilimumab in liver cancers also show modulation of the TME with increased immune infiltration and activation of type I IFN signatures in interim ASCO 2024 results (NCT04535229; PERIO-02 study, J Clin Oncol, 2024).

The combination of STING agonists with VEGF/VEGFR inhibition represents an emerging immuno-vascular strategy that remains largely at the preclinical stage. A key preclinical study showed that combining a STING agonist with an anti-angiogenic treatment resulted in significant tumor regression in murine models, providing the basis for this immuno-vascular synergy [217]. Evidence suggests that STING activation can induce inflammation often restricted to the tumor stroma, whereas anti-VEGF therapy improves immune cell infiltration. Their combination therefore appears relevant for TME reprogramming strategies. Ongoing developments are moving toward triplet approaches integrating STING agonists, VEGF/VEGFR inhibition, and radiotherapy or ICD-inducing chemotherapy, aiming to achieve coordinated amplification of innate immunity, vascular access, and adaptive antitumor responses.

#### Targeting stromal and immunosuppressive barriers

In stromal-rich tumors, particularly pancreatic cancer, ECM remodeling constitutes a major physical barrier to CD8⁺ T-cell infiltration. CAFs produce collagen, fibronectin, and immunosuppressive factors such as TGF-β and CXCL12, thereby creating a highly restrictive microenvironment. Targeting TGF-β or the CXCL12/CXCR4 axis can reduce spatial immune exclusion and improve intratumoral T-cell distribution.

Targeting the TGF-β pathway in combination with immunotherapies (mainly anti–PD-1/PD-L1) represents an emerging strategy aimed at overcoming a key mechanism of immune resistance associated with an immunosuppressive and “immune-excluded” TME. A phase I/Ib study showed that the anti–TGF-β antibody SAR439459, administered alone or in combination with the anti–PD-1 antibody Cemiplimab, induced increases in IFN-γ and CXCL10 signatures, along with immune reprogramming characterized by expansion of NK and T cells, and in some cases a conversion from an “immune-excluded” to an “immune-inflamed” phenotype [218]. Similarly, a phase I trial of the selective TGF-β1 inhibitor SRK-181 (Linavonkibart) combined with Pembrolizumab in patients resistant to anti–PD-1 therapy showed early clinical signals of activity in refractory solid tumors, supporting the rationale for targeting TGF-β1 to restore ICI sensitivity [219]. In addition, bispecific approaches such as LBL-015 presented at ASCO 2024, combining PD-1 inhibition and TGF-β receptor blockade (NCT05107011, J Clin Oncol, 2024), demonstrated feasibility and early efficacy signals with modulation of the tumor immune microenvironment. Innovative strategies such as oncolytic viruses armed with a TGF-β trap (AdAPT-001) combined with ICIs further confirm the value of neutralizing TGF-β to enhance lymphocyte infiltration and antitumor immunity [220]. Likewise, bifunctional agents such as bintrafusp alfa, combining TGF-β and PD-L1 blockade, have shown clinical activity in combination with chemotherapy in advanced cancers, highlighting the interest in integrating cytotoxic therapy with immunotherapy and suppression of tumor tolerance pathways [221]. Targeting the TGF-β pathway in combination with VEGF/VEGFR inhibition and immunotherapy represents an emerging strategy aimed at simultaneously reprogramming immunosuppressive and vascular compartments of the TME. A key clinical proof-of-concept comes from TU2218, a dual inhibitor targeting both TGF-βR1 and VEGFR2, evaluated in phase I studies as monotherapy and in combination with Pembrolizumab in advanced solid tumors. These studies showed acceptable tolerability, biomarker modulation, and early antitumor activity signals, supporting the feasibility of dual immuno-vascular targeting (Phase 1a: NCT05204862; Phase 1b: NCT05784688, [222]). Nevertheless, several clinical trials targeting TGF-β pathways have shown limited efficacy, emphasizing the complexity of TGF-β biology and the risk of simultaneously affecting tumor-suppressive and tumor-promoting functions.

Targeting the CXCL12–CXCR4 axis in combination with immunotherapies represents an emerging strategy to overcome immune exclusion in stromal-rich tumors, including microsatellite-stable CRC, pancreatic cancer, and glioblastoma [223, 224]. The key clinical study is the OPERA trial, a phase I/II evaluation of the CXCL12 inhibitor NOX-A12 in combination with Pembrolizumab. This trial demonstrated that CXCL12 blockade induces TME reprogramming with increased intratumoral T-cell infiltration and modulation of cytokine signatures, while combination with anti–PD-1 led to disease stabilization in a subset of heavily pretreated patients, albeit without major objective responses [223]. Finally, inhibition of the CXCR4 axis using Plerixafor combined with anti–PD-1 therapy (Cemiplimab) in metastatic pancreatic cancer confirmed the ability of this strategy to enhance intratumoral T-cell recruitment in a phase II study, but also revealed compensatory immunosuppression through expansion of macrophages, limiting overall clinical efficacy ( [225]; NCT04177810).

Targeting the CXCL12–CXCR4 axis in combination with VEGF/VEGFR inhibition also represents a TME reprogramming strategy. The most informative clinical data come from combination studies involving CXCR4 inhibitors and anti-VEGF agents, notably a phase I/biomarker study evaluating Plerixafor plus Bevacizumab in glioblastoma, which demonstrated feasibility of dual targeting, modulation of myeloid and vascular compartments, and increased immune cell infiltration, although without a major survival benefit ( [226], NCT01339039). In addition, translational and clinical studies in glioblastoma have provided direct evidence supporting dual CXCL12 and VEGF targeting, particularly in the GLORIA trial, where the combination of the CXCL12 aptamer NOX-A12 and Bevacizumab showed reduced tumor perfusion and improved survival-related parameters compared with anti-VEGF alone ( [227, 228]; NCT04121455).

Targeting the CXCL12–CXCR4 axis in combination with chemotherapy and ICIs represents a TME reprogramming strategy extensively investigated in “cold” solid tumors, particularly pancreatic cancer and microsatellite-stable CRC. The reference study is COMBAT, a phase IIa trial evaluating the CXCR4 antagonist BL-8040 (Motixafortide) in combination with Pembrolizumab and Irinotecan/5-FU chemotherapy in metastatic PDAC. This study demonstrated increased intratumoral CD8⁺ T-cell infiltration, decreased myeloid suppressor cells, and a disease control rate of approximately 77% in the chemo-immunotherapy cohort, suggesting synergy between cytotoxicity, stromal modulation, and immune activation [229].

#### Inducing TLS to reorganize antitumor immune infiltrates

Strategies for inducing TLS generally follow two complementary paradigms: a “ligand-first” approach that reactivates lymphoid organogenesis signals (e.g., LTβR/LIGHT and CXCL13, CCL19/CCL21), and a “niche-first” approach that establishes locally permissive microenvironments. Both aim to progressively and durably convert immunologically “cold” tumors into immune-organized, TLS-enriched microenvironments.

Delivery of cytokines and chemokines is most effective when it enables the remodeling of HEVsand the organization of lymphoid structures, rather than simply increasing lymphocyte influx. A vascular-targeted LIGHT-VTP fusion protein has been shown to induce HEV remodeling and TLS formation in pancreatic neuroendocrine tumors and Lewis lung carcinoma, with synergistic activity in combination with PD-1/CTLA-4 blockade [138]. Similarly, concomitant local activation of LTβR and LTα enhanced TLS formation and promoted development of mature follicular DC and fibroblastic reticular cell networks, supporting the concept that coordinated delivery of organizational and chemokine signals may favor TLS maturation [230]. From a translational perspective, these approaches are attractive but may be associated with off-target inflammation and difficult-to-control chemokine gradients. Therefore, spatially controlled, imaging-guided, and sustained-release approaches appear more clinically promising, although standardization and real-time safety monitoring remain major challenges.

Biomaterials and tissue engineering approaches offer an opportunity to achieve localized and sustained presentation of immune-organizing signals. A microporous gelatin methacryloyl scaffold implanted into post-resection cavities and loaded with antigens and CpG promoted HEV formation and B/T-cell aggregation. In combination with PD-1 blockade, it reduced recurrence and metastasis [231]. Ferritin–resilin hydrogels carrying CP05 peptides enabled efficient delivery of CXCL13/CCL21-enriched exosomes, inducing ectopic lymphoid structures at subcutaneous sites and increasing complete responses to PD-1 blockade [232]. Covalent organic frameworks with photothermal and aggregation-induced emission properties induced CXCL10 and CCL19 production, promoted TLS formation, and synergized with anti-CTLA-4 in MC38 and 4MOSC1 tumor models [233]. COF-306@FM biomimetic constructs further enhanced immune-cell homing cues and TLS formation, strengthening systemic antitumor immunity in combination with PD-L1 blockade [234]. These material-based strategies are particularly suited to post-resection sites and accessible lesions, but clinical translation will depend on demonstrated biocompatibility, biodegradability, scalable manufacturing, and regulatory clarity.

Innate immune agonists—particularly STING and TLR platforms—provide a rapid “priming” mechanism to reshape local inflammation toward TLS neogenesis and maturation. The ZCCG hydrogel (Zn^2^⁺/chitosan/CpG) activates both cGAS–STING and TLR9 pathways, increases CXCL10, CCL19, and CCL21 expression, promotes HEV formation, and synergizes with PD-1 blockade in breast cancer and melanoma models [235]. In parallel, the TLR4 agonist OK432 combined with tumor antigens activates LTβR signaling and promotes TLS formation [236]. These agonists can rapidly convert “cold” TMEs into TLS-permissive environments, although systemic cytokine toxicity and a narrow therapeutic window currently limit their clinical use.

Several studies indicate that ICIs also influence TLS formation. Mechanistic work in RIP1-Tag5 and MC38 models shows that combined PD-1/CTLA-4 blockade increases CXCL13 and CCL19 levels and promotes TLS assembly [138]. In PDAC, KRAS inhibition cooperates with CTLA-4 blockade to remodel the TME and promote TLS maturation. Clinically, in HCC, ICIs are associated with increased intratumoral TLS density and improved response rates and recurrence-free survival [148]. Thus, ICIs not only release immune checkpoints but are also associated with changes in the chemokine and cellular environment that may favor TLS neogenesis and maturation, although their causal role remains debated.

Conventional therapies can also contribute to TLS formation. In ovarian cancer, neoadjuvant platinum–taxane therapy promotes antigen release and induces germinal center–like TLS [142]. In nasopharyngeal carcinoma, Gemcitabine–Cisplatin increases B-cell infiltration and modulates T follicular helper cells, generating TLS-like structures [237]. FOLFOX-based treatment in HCC also significantly enhances TLS formation [238]. However, these effects are highly dependent on treatment schedules and may be limited by lymphodepletion.

Oncolytic viruses selectively lyse tumor cells and release immunostimulatory signals. In multiple models, oncolytic herpes viruses enhance the CXCL10–CXCR3 axis, recruit CD8⁺ TCF1⁺ T cells, and induce TLS formation with B-cell aggregation, effects further enhanced by PD-1 blockade [153].

A proposed integrative framework involves priming with ICD-inducing therapies to initiate antigen release and inflammatory signaling, followed by LTβR/LIGHT- and CXCL13/CCL19/CCL21-dependent mechanisms to organize lymphoid niches, with immune checkpoint inhibition supporting their functional maturation. VEGF blockade-mediated vascular normalization may be intercalated to improve immune cell access and facilitate TLS development. Importantly, TLS formation alone does not necessarily imply effective antitumor immunity, as their functional impact depends on their maturation state, cellular composition, vascular organization, and capacity to support local antigen presentation.

## Future directions

**Fig. 6:**
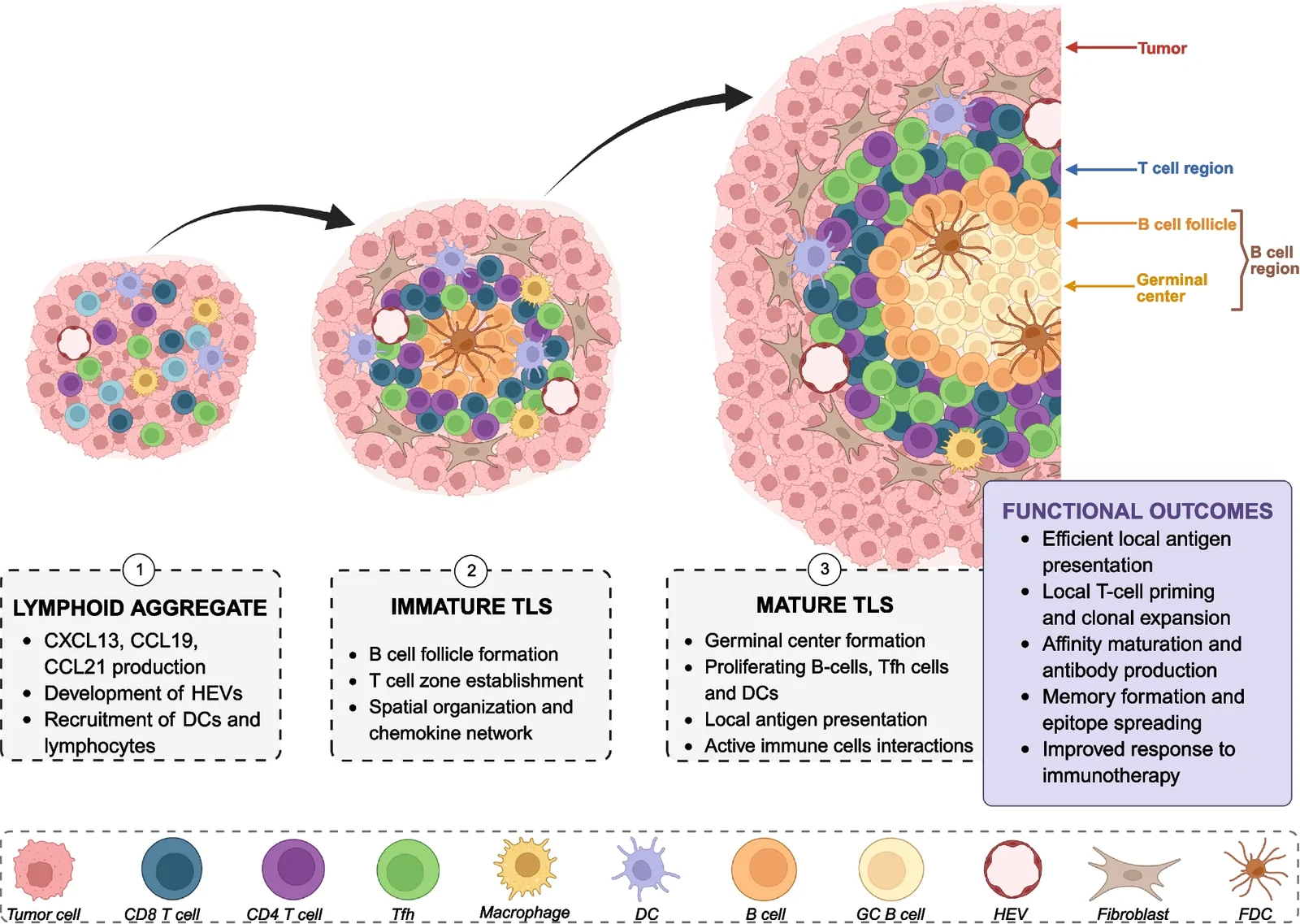
Framework for engineering the tumor microenvironment to convert immunologically “cold” tumors into “hot” tumors. The proposed strategy comprises six complementary steps: (1) Comprehensive characterization of the TME to identify the dominant mechanisms of immune resistance using spatial and single-cell technologies; (2) remodeling of physical barriers, including abnormal vasculature and stromal components, to facilitate immune cell infiltration; (3) induction of local inflammation through activation of innate immune sensing pathways and inflammatory cytokine production; (4) recruitment of CXCR3⁺ CD8⁺ T cells by enhancing the expression of CXCL9, CXCL10, and CXCL11; (5) restoration of T-cell effector function through immune checkpoint blockade and co-stimulatory signaling; and (6) establishment of durable antitumor immunity via TLS formation, memory T-cell generation, and epitope spreading

Despite major advances in understanding the mechanisms that limit CD8⁺ T cell infiltration into tumors, translating this knowledge into durable clinical benefit remains challenging. Importantly, several approaches that successfully increased CD8⁺ T-cell infiltration in preclinical models have shown limited clinical efficacy, highlighting a major translational challenge: immune exclusion is not controlled by a single dominant pathway but results from the convergence of multiple adaptive resistance mechanisms. Future progress will likely depend on a shift from single-pathway interventions toward integrated, spatially informed, and adaptive strategies capable of simultaneously addressing stromal, vascular, chemokine-related, and immunosuppressive barriers. A key upcoming challenge will be the use of sequential therapies, which may serve not only to enhance CD8⁺ T cell infiltration but also to control their spatial and temporal organization within the tumor tissue (Fig. 6). Indeed, different therapeutic classes act on distinct levels of this hierarchical organization. Thus, “therapeutic sequencing” should be viewed as a spatiotemporal TME reprogramming strategy, in which each drug targets a specific biological barrier. The ultimate goal is to achieve a coordinated, durable, and self-amplifying local immune response. This dynamic perspective requires considering cancer as an evolving system whose immunological organization is directly shaped by treatment timing. These therapeutic sequences are part of the broader concept of microenvironmental reprogramming, which increasingly relies on new insights into the spatiotemporal biology of the TME enabled by emerging technologies.

A first major direction concerns the development of spatial immuno-oncology, enabled by technologies such as spatial transcriptomics, single-cell sequencing, and multiplex imaging. These approaches now allow high-resolution mapping of CD8⁺ T cell exclusion, distinguishing true “immune-desert” regions from “immune-excluded” phenotypes, where T cells are trapped within the stroma or perivascular niches. This spatial characterization will be essential to identify dominant resistance mechanisms in each tumor lesion and to guide rational rather than empirical therapeutic combinations.

Second, the field is moving toward personalized medicine strategies. Patients present tumors with heterogeneous levels of fibroblast activation, ECM deposition, vascular dysfunction, and immune cell sequestration. Future approaches will therefore increasingly rely on stratifying the TME in order to select the most relevant therapeutic combinations.

A third emerging direction is the design of adaptive multi-step therapeutic frameworks, in which treatments are administered according to biological logic rather than in uncontrolled simultaneous combinations. Optimizing both the sequence and the timing of these steps will likely be as important as selecting the therapeutic agents themselves.

What are the barriers to implementing sequential strategies? Delivering three drugs from different therapeutic classes remains a major challenge, even though real programs are being developed. Toxicity management is a key issue: combining multiple agents increases toxicity not only additively but sometimes synergistically. In addition, evaluating the efficacy of sequential administration of two or three therapies is complex, as the number of variables increases substantially, including drug choice, combinations, order of administration, dose, and treatment duration. Consequently, the number of patients required to test these different hypotheses and identify optimal sequencing strategies may become very large and logistically challenging. Moreover, beyond their biological rationale, the true translational potential of strategies aimed at improving CD8⁺ T-cell infiltration will depend on their ability to provide durable clinical benefit, acceptable toxicity, and applicability across heterogeneous tumor contexts. Approaches such as vascular normalization and immune checkpoint combinations have already entered clinical practice, whereas strategies involving TLS induction, chemokine receptor engineering, stromal remodeling, or innate immune agonists remain largely experimental. Importantly, increasing CD8⁺ T-cell density alone is unlikely to be sufficient, as therapeutic success requires coordinated improvement of immune recruitment, spatial organization, persistence, and effector function. Future clinical development will therefore require robust predictive biomarkers to identify which immune-excluded tumors are most likely to respond to specific microenvironmental interventions.

An important lesson emerging from both preclinical and clinical studies is that no single intervention appears sufficient to durably restore anti-tumor immunity. Although many approaches successfully increase CD8⁺ T-cell infiltration, this does not necessarily translate into sustained clinical responses because infiltrating lymphocytes may remain spatially restricted, metabolically impaired, or functionally exhausted. Likewise, improving T-cell function without correcting defects in trafficking or tissue accessibility is unlikely to generate durable benefit. These observations support the development of integrated therapeutic strategies targeting multiple levels of the cancer-immunity cycle in a coordinated and temporally optimized manner.

Overall, the future of strategies aimed at improving CD8⁺ T cell infiltration lies in the convergence of spatial biology, systems immunology, and rational combination therapy design. The central goal is no longer solely to activate T cells, but to engineer tumor ecosystems capable of enabling, guiding, and sustainably supporting their infiltration and effector function.

## Data Availability

No new data were generated in this study.

## Abbreviations

APC: Antigen Presenting Cell
ATP: Adenosine Triphosphate
ATRA: All-Trans Retinoic Acid
CAF: Cancer-Associated Fibroblast
cAMP: Cyclic Adenosine Monophosphate
CAR: Chimeric Antigen Receptor
CCL: C–C motif chemokine Ligand
CCR: C–C motif chemokine Receptor
CD: Cluster of Differentiation
cGAS: Cyclic GMP-AMP Synthase
CpG: Cytosine-Phosphate-Guanine
CRC: Colorectal Cancer
CTLA-4: Cytolytic T-Lymphocyte-Associated protein 4
CX3CL: C-X3-C motif chemokine Ligand
CX3CR: C-X3-C motif chemokine Receptor
CXCL: C-X-C motif chemokine Ligand
CXCR: C-X-C motif chemokine Receptor
DAMP: Damage-Associated Molecular Patterns
DC: Dendritic Cell
DNA: Deoxyribonucleic Acid
DNMT1: DNA methyltransferase 1
ECM: Extracellular Matrix
ENTPD1: Ectonucleoside Triphosphate Diphosphohydrolase-1
EZH2: Enhancer Of Zeste Homolog 2
FAP: Fibroblast Activation Protein
FDA: Food and Drug Administration
FLT3L: Fms-Related Tyrosine Kinase 3 Ligand
Gal9: Galectin-9
GZMK: Granzyme K
HCC: Hepatocellular Carcinoma
HEV: High Endothelial Venule
HIF-1a: Hypoxia-Inducible Factors Alpha
HMGB1: High Mobility Group Box 1
HSP: Heat Shock Proteins
ICAM-1: Intercellular Adhesion Molecule 1
ICD: Immunogenic Cell Death
ICI: Immune Checkpoint Inhibitor
ID: Differentiation Inhibitors
IFN: Interferon
IL: Interleukin
ILC3: Innate Lymphoid Cell Type 3
KLF2: Krüppel-like Factor 2
KRAS: Kirsten Rat Sarcoma Virus Oncogene Homologue
LFA-1: Lymphocyte Function-associated Antigen-1
LIGHT: Tumor Necrosis Factor Superfamily Member 14
LT-a/b: Lymphotoxin Alpha/Beta Receptor
MDSC: Myeloid-Derived Suppressor Cell
MEK: Mitogen-Activated Protein Kinase
MHC: Major Histocompatibility Complex
NK: Natural Killer
NSCLC: Non-Small Cell Lung Cancer
NT5E: 5′-Nucleotidase Ecto
PAMP: Pathogen-Associated Molecular Patterns
PD-1: Program Death 1
PDAC: Pancreatic Ductal Adenocarcinoma
PD-L1: Program Death Ligand 1
PGE_2_: Prostaglandin E_2_
RNA: RiboNucleic Acid
SMAD: Suppressor of Mothers Against Decapentaplegic
STING: Stimulator of Interferon Genes
TAM: Tumor-Associated Macrophage
T-bet: T-box Expressed in T cells
Tbx-21: T-Box Transcription Factor T-bet
TCF: T Cell Factor
TCR: T Cell Receptor
TDLN: Tumor-Draining Lymph Nodes
Tfh: Follicular Helper T Cell
TGF-b: Transforming Growth Factor Beta
TKI: Tyrosine Kinase Inhibitors
TLR: Toll Like Receptors
TLS: Tertiary Lymphoid Structures
TME: Tumor Microenvironment
TNF: Tumor Necrosis Factor
TP53: Tumor Protein p53
Treg: Regulatory T Cells
TRM: Tissue-Resident Memory T Cells
VCAM-1: Vascular Adhesion Molecule 1
VEGF: Vascular Endothelial Growth Factor
VEGFR: Vascular Endothelial Growth Factor Receptor
VLA-4: Very Late Antigen 4
Zn^2^⁺: Zinc
α-SMA: Alpha-smooth muscle actin
